# Serotonergic neurons in the ventral nerve cord of Chilopoda – a mandibulate pattern of individually identifiable neurons

**DOI:** 10.1186/s40851-017-0070-y

**Published:** 2017-07-04

**Authors:** Andy Sombke, Torben Stemme

**Affiliations:** 1 University of Greifswald, Zoological Institute and Museum, Cytology and Evolutionary Biology, Soldmannstrasse 23, 17487 Greifswald, Germany; 2 Division of Cell Biology, University of Veterinary Medicine Hannover, Bischofsholer Damm 15/102, 30173 Hannover, Germany; 3 Current address: University of Ulm, Institute for Neurobiology, Helmholtzstraße 10/1, 89081 Ulm, Germany

**Keywords:** 5HT, Tetraconata, Miracrustacea, Mandibulata, Arthropoda, Neurophylogeny, Evolution, VNC

## Abstract

**Background:**

Given the numerous hypotheses concerning arthropod phylogeny, independent data are needed to supplement knowledge based on traditional external morphology and modern molecular sequence information. One promising approach involves comparisons of the structure and development of the nervous system. Along these lines, the morphology of serotonin-immunoreactive neurons in the ventral nerve cord has been investigated in numerous tetraconate taxa (Crustacea and Hexapoda). It has been shown that these neurons can be identified individually due to their comparably low number, characteristic soma position, and neurite morphology, thus making it possible to establish homologies at the single cell level. Within Chilopoda (centipedes), detailed analyses of major branching patterns of serotonin-immunoreactive neurons are missing, but are crucial for developing meaningful conclusions on the homology of single cells.

**Results:**

In the present study, we re-investigated the distribution and projection patterns of serotonin-immunoreactive neurons in the ventral nerve cord of three centipede species: *Scutigera coleoptrata*, *Lithobius forficatus*, and *Scolopendra oraniensis*. The centipede serotonergic system in the ventral nerve cord contains defined groups of individually identifiable neurons. An anterior and two posterior immunoreactive neurons per hemiganglion with contralateral projections, a pair of ipsilateral projecting lateral neurons (an autapomorphic character for Chilopoda), as well as a postero-lateral group of an unclear number of cells are present in the ground pattern of Chilopoda.

**Conclusions:**

Comparisons to the patterns of serotonin-immunoreactive neurons of tetraconate taxa support the homology of anterior and posterior neurons. Our results thus support a sister group relationship of Myriapoda and Tetraconata and, further, a mandibulate ground pattern of individually identifiable serotonin-immunoreactive neurons in the ventral nerve cord. Medial neurons are not considered to be part of the tetraconate ground pattern, but could favor the ‘Miracrustacea hypothesis’, uniting Remipedia, Cephalocarida, and Hexapoda.

## Background

The morphology of serotonin-immunoreactive (5HT-ir) neurons in the ganglia of the arthropod ventral nerve cord (VNC) caught the attention of scientists over the past 30 years (summarized in [1]). It has been proposed that these neurons are a suitable character set for phylogenetic comparisons due to the following characteristics: (1) Only a small number of neurons possessing serotonin (5HT) as neurotransmitter is distributed in the nervous system, (2) intra- and interspecific serial homology provide opportunities to reconstruct evolutionary scenarios, and (3) numerous studies have described these neurons in a great diversity of arthropod species. However, since the availability of specific antibodies against 5HT [2, 3], approaches for the identification of these neurons has mainly focused on Tetraconata (Crustacea and Hexapoda), starting with investigations in lobsters [4] and cockroaches [5]. The first comparative studies pointing out striking morphological similarities of certain neuron types were initially restricted to winged insects, such as dragonflies, cockroaches, grasshoppers, and flies [6], inferring homologies at single cell level [7]. The homology of individually identifiable 5HT-ir neurons in insects was strongly supported by developmental studies in grasshopper and the fruit fly, demonstrating that these neurons are derived from the same neuronal progenitor cell, the neuroblast 7-3 [5, 8, 9].

To date, an impressive body of literature describing the serotonergic transmitter system in numerous crustacean and hexapod species has accumulated [4–6, 10–20]. Along these lines, Harzsch [21, 22] reconstructed ground patterns for major tetraconate lineages, demonstrating the potential of individually identifiable neurons as independent characters for evolutionary considerations. Recently, corresponding studies have been undertaken for the crustacean taxa Cephalocarida [23] and Remipedia [24], as well as the hexapod taxa Zygentoma and Archaeognatha [1], including an update on specific ground patterns (sensu [22]). Although a wealth of tetraconate taxa have been investigated in great detail, our knowledge on more basal arthropod lineages, i.e., Chelicerata and Myriapoda, is limited. Initial insights into the distribution of 5HT-ir neurons in the VNC of Chelicerata and Myriapoda indicated that the patterns of these cells differ remarkably from those described in Tetraconata [22, 25, 26]. Recently it was shown that Pycnogonida (Chelicerata) possess individually identifiable 5HT-ir neurons, although homologization of these neurons to those in tetraconate representatives remains challenging [27].

In general, 5HT-ir neurons in Tetraconata are located in anterior as well as posterior positions in VNC ganglia. In recent studies on Cephalocarida [23], Remipedia [24], as well as basal insects [1], yet another group of neurons has been described in a medial position. In order to gain deeper insights into the phylogenetic significance of these data for Tetraconata, it is important explore the situation in Myriapoda, which is the proposed sister taxon to Tetraconata [28–30]. Myriapods play a crucial role in considerations of evolutionary transformations of arthropod nervous systems [29, 31–33]. However, while to date the study by Harzsch [22] is the only to describe 5HT-ir neurons in the myriapod ventral nerve cord, the projection pattern of these neurons remained unresolved. Due to recent accounts and resulting controversies in interpreting these data, a detailed re-examination of myriapod taxa has been demanded [1, 23, 27], as information on basal mandibulate taxa is crucial for our understanding of the phylogenetic value of 5HT-ir neurons. In this context, two hypotheses concerning the tetraconate ground pattern have been proposed. In the first, postulated by Harzsch [22], two anterior and two posterior bipolar neurons are present, one neurite projecting contralaterally, the other ipsilaterally. The second hypothesis, by Stegner et al. [23], which takes into account newer data from Remipedia [24] and Cephalocarida [23], suggests a more complex scenario. Here, the tetraconate ground pattern comprises anterior neurons with unclear projection pattern (ipsi- or contralateral), a posterior pair that possesses contralateral projections, and at least one medially positioned neuron with an ipsilateral projection. The exact number of medial neurons for this ground pattern is unclear due to insufficient information concerning outgroup taxa such as Myriapoda and Chelicerata (see [23]). To add further data and contribute to the question whether a common pattern of individually identifiable serotonergic neurons in Chilopoda and Tetraconata exists, we revisited the 5HT-ir system in the VNC of three centipede species from Scutigeromorpha, Lithobiomorpha, and Scolopendromorpha in order to reveal the distribution and projection pattern of 5HT-ir neurons in their VNC.

## Methods

### Experimental animals

**Fig. 1:**
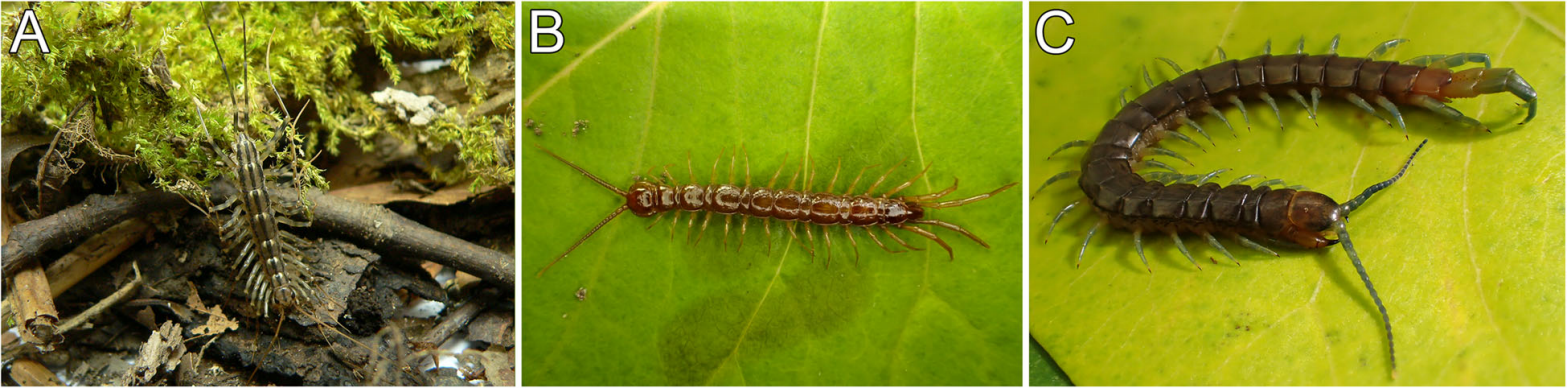
The three centipede species investigated. **a** *Scutigera coleoptrata.* **b** *Lithobius forficatus.* **c** *Scolopendra oraniensis.* Originals

Adult specimens of *Scutigera coleoptrata* (Fig. 1a) and *Scolopendra oraniensis* (Fig. 1c) were collected on the Balearic island of Ibiza (Spain), mainly in pine forests. Adult specimens of *Lithobius forficatus* (Fig. 1b) were collected in Greifswald (Germany) under stones and deadwood. All specimens were kept in plastic boxes at room temperature. All procedures in this investigation were conducted in compliance with international and institutional guidelines, including the guidelines for animal welfare as laid down by the German Research Foundation (DFG).

### Preparation and fixation

Living specimens were cold-anesthetized for several minutes and prefixed for 10 min at room temperature in 4% paraformaldehyde (PFA) (Sigma Aldrich, #P6148, St. Louis, MO) in sodium hydrogen phosphate buffer (PBS, 0.1 M, pH 7.4; chemicals obtained from Carl Roth, Karlsruhe, Germany). Subsequently, specimens were decapitated and the VNC was dissected with fine forceps and fixed in 4% PFA dissolved in PBS over night at 4 °C.

Four specimens of each species were treated as whole mounts, two additional specimens of each species were further processed for vibratome sectioning. For the latter, preparations were washed in PBS, overlaid with Poly-L-Lysin (Sigma Aldrich, #P8920) for several minutes and embedded in 4% agarose (Sigma Aldrich, #A9414) dissolved in PBS at approximately 40 °C. After cooling to room temperature, the trimmed agarose-blocks were sectioned horizontally (80 μm) using a Microm HM 650 V vibratome (Thermo Scientific, Eugene, OR).

### Immunocytochemistry

All steps described below were performed on a shaker with smooth agitation at room temperature, if not otherwise stated. Whole mount preparations were washed in PBS and incubated for 1 h in PBS with 0.5% Triton X-100 (Sigma Aldrich, #X100) (PBS-TX 0.5%), followed by a blocking step in 5% bovine serum albumin (BSA) (Sigma Aldrich, #A2153) in PBS-TX 0.5% with 0.05% sodium azide (Carl Roth, #K305) at least 4 h. Subsequently, preparations were incubated in the primary antibody rabbit-anti-5HT (Immunostar, Hudson, WI, USA, #20080) diluted 1:1000 in blocking solution for 48 h. After four washing steps in PBS-TX 0.5% for 30 min each, the preparations were incubated in the secondary antibody goat-anti-rabbit conjugated to Alexa Fluor 488 (Invitrogen, #A11008; Carlsbad, CA, USA; dilution: 1:500) plus 0.1% of the nuclear marker bisBenzimide H 33258 (Sigma Aldrich, #14530) for 48 h. Preparations were rinsed for 2 h in four changes of PBS-TX 0.5% and in a final step in PBS, followed by incubations in glycerol (Carl Roth, #3783) / PBS 1:1 and glycerol/PBS 9:1. Finally, preparations were mounted on glass slides in glycerol/PBS 9:1 with 2.5% Dabco (Carl Roth, #0718) as anti-fading agent.

Immunocytochemistry on vibratome sections underwent the same procedure with the following differences: A permeabilization step was omitted and the concentration of Triton X-100 has been reduced to 0.3%. Incubations in first and secondary antibody solutions lasted 24 h and preparations were not cleared with glycerol, but mounted in Mowiol (Merck, #475904, Darmstadt, Germany).

### Antibody characterization

The polyclonal anti-5HT antibody (rabbit, ImmunoStar, #20080) was raised against 5HT coupled to BSA with paraformaldehyde. The same antibody has been applied in several studies on arthropod nervous systems [23, 27, 34–37]. Preadsorption controls showed that the antibody does not recognize a BSA-epitope instead of 5HT (see datasheet manufacturer; [34, 35]). In control experiments for nonspecific bindings of secondary antisera, we replaced the primary antibody by blocking solution, resulting in the absence of labeling.

### Image acquisition

Sections were examined with a Nikon eclipse 90i microscope (Tokyo, Japan) and a Leica SP5 II confocal microscope (cLSM) (Wetzlar, Germany). Z-series were processed with NIH ImageJ, v. 1.44 (Rasband WS, ImageJ, U.S. National Institutes of Health, Bethesda, MD, http://rsb.info.nih.gov/ij/), producing maximum projections. Images were processed in Adobe Photoshop 6.0 (San Jose, CA) using global contrast and brightness adjustment features, as well as black and white inversion.

## Results

### Morphology of the ventral nerve cord of Chilopoda

**Fig. 2:**
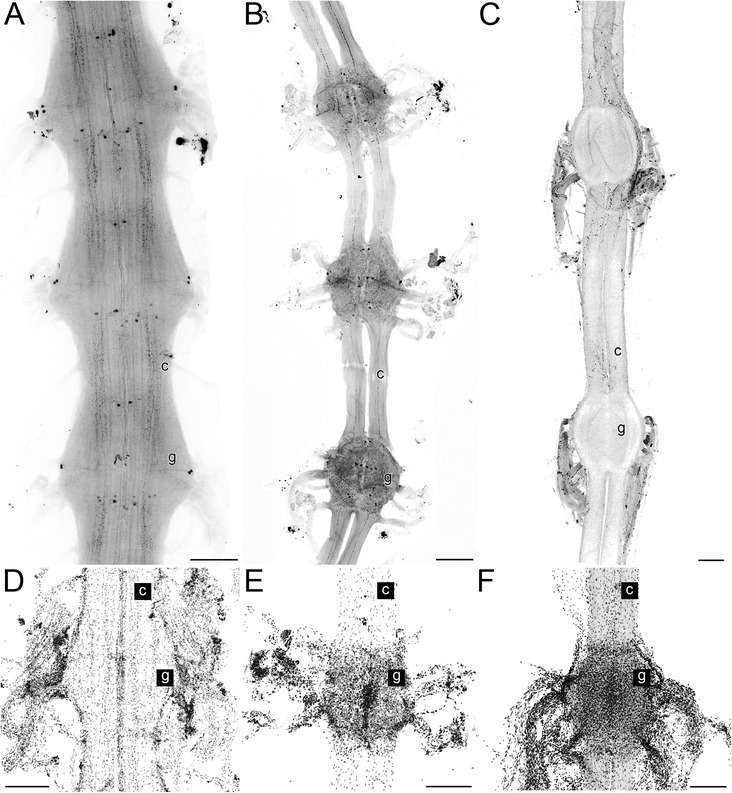
Centipede ventral nerve cord ganglia (whole mounts) and nuclear labeling of single ganglia. Maximum projections of cLSM stacks, anterior to the top. **a** Ganglia 9–11 of the ventral nerve cord of *Scutigera coleoptrata* with individually identifiable 5HT-ir neurons. Note the fusion of connectives and segmental organization of leg nerves. **b** Ganglia of the ventral nerve cord of *Lithobius forficatus* with individually identifiable 5HT-ir neurons. In contrast to *S. coleoptrata*, the long connectives are separated. **c** Ganglia of the ventral nerve cord of *Scolopendra oraniensis*. As whole mount labeling against serotonin failed, no individual neurons are visible. Note the long connectives. **d** Nuclear labeling in a ganglion of *S. coleoptrata.* Note that the density of somata surrounding the ganglia (g) is equal to that around the fused connectives (c). **e** Nuclear labeling in a ganglion of *L. forficatus.* Note that the density of somata surrounding the ganglion (g) is much higher than around the connectives (c). **f** Nuclear labeling in a ganglion of *S. oraniensis.* Note that the density of somata surrounding the ganglion (g) is much higher than around the connectives (c). Scale bars = 250 μm

In slightly overexposed cLSM analysis, the general morphology of the VNC is clearly visible. In *Scutigera coleoptrata*, the VNC appears as a more or less contiguous fused cord, as ganglia are completely fused medially (Fig. 2a) and separate connectives are absent. In contrast, separate connectives are present in *Lithobius forficatus* (Fig. 2b) and *Scolopendra oraniensis* (Fig. 2c). In *Scutigera coleoptrata* and *Lithobius forficatus*, individual 5HT-ir somata are detectable in whole mounts (Fig. 2a, b). Whole mount experiments with *Scolopendra oraniensis* failed; thus, in these preparations no 5HT-ir somata or neurites are detectable (Fig. 2c). In addition, somata distribution differs in the investigated species. While in *Scutigera coleoptrata*, somata are more or less evenly distributed (also in the region of fused connectives; Fig. 2d), in *Lithobius forficatus* and *Scolopendra oraniensis*, only a few somata are associated with the connectives (Fig. 2e, f).

### *Serotonin immunoreactivity in the ventral nerve cord of* Scutigera coleoptrata

**Fig. 3:**
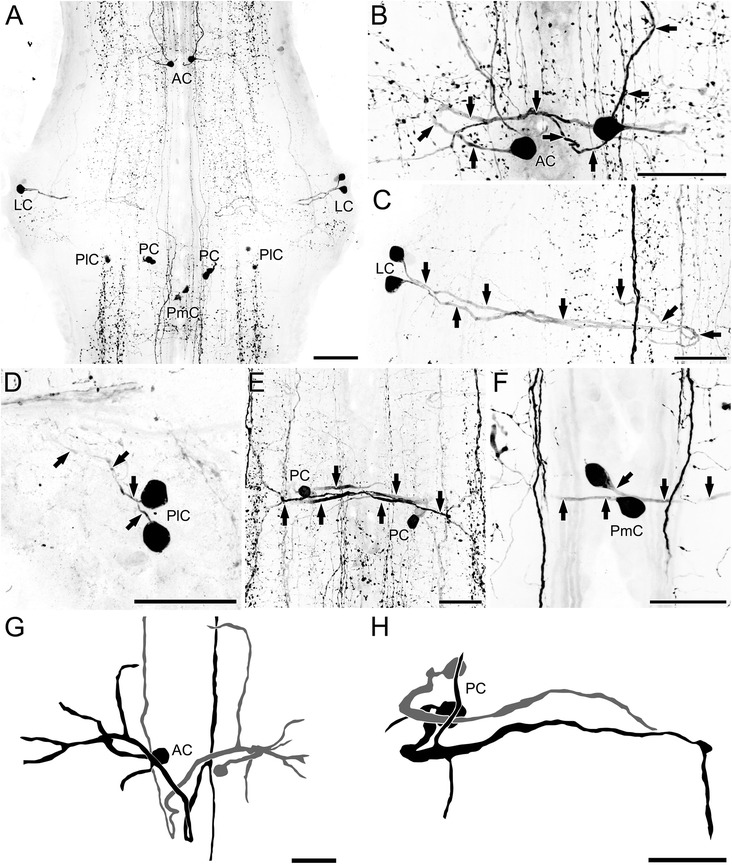
Distribution of 5HT-ir neurons in the VNC of *Scutigera coleoptrata*. All neurons were detected in every ganglion of all preparations. **a** These individually identifiable neurons can be distinguished according to somata positions within the ganglia into anterior (AC), lateral (LC), postero-lateral (PlC), posterior (PC), and postero-medial cells (PmC). **b** The AC project their neurites contralaterally (indicated for the left neuron by arrows). **c** The LC project their neurites medially towards the midline, but do not cross the latter (arrows). **d** Neurites of the PlC could only be traced for a short distance, projecting antero-laterally (arrows). **e** The pair of PCs sent their processes contralaterally (arrows). **f** The PmCs project laterally (arrows). As these cells are situated near the midline it was not always clear whether they project ipsi- or contralaterally. **g** Reconstructions of ACs. **h** Reconstructions of PCs. Compare also Fig. 6. Scale bars: A = 100 μm; B–H = 50 μm

Generally, each trunk ganglion of the VNC contains the same number and arrangement of 5HT-ir neurons, indicating serial homology (Fig. 2a). Ganglia are characterized by two longitudinal immunoreactive bundles containing few antero-posterior directed neurites and exhibit patchy immunoreactivity (Figs. 2a and 3a). In addition, a thin medially positioned bundle of immunoreactive neurites passes through the hemiganglia in antero-posterior orientation (Fig. 3a, e). All 5HT-ir neurons can be identified individually and divided into five groups according to their spatial soma position (Figs. 2a and 3a–f). In each hemiganglion a single anterior neuron (AC, Fig. 3a, b, g), two lateral neurons (LC, Fig. 3a, c), two postero-lateral neurons (PlC, Fig. 3a, d), two posterior neurons (PC, Fig. 3a, e, h), and a single postero-medial neuron (PmC, Fig. 3a, f) are present. All of these neurons are unipolar.

The primary neurite of the anterior neuron projects laterad (Fig. 3b, g). After a short distance the course is reversed to the contralateral hemiganglion (Fig. 3b, g). In the midline of the ganglion, the neurites of both heterolateral neurons cross each other (Fig. 3b, g). In the contralateral hemiganglion, the neurite extends slightly posterior before it bifurcates and the resulting branches project anteriad and posteriad into the adjacent connectives/ganglia, indicating an ascending pathway (Fig. 3a, b, g). Neurites of the lateral pair of neurons project medially, passing the main longitudinal immunoreactive bundle (Fig. 3c). Near the midline, they turn 180° and project antero-laterad, joining the ipsilateral 5HT-ir bundle (Fig. 3c). Neurites of the postero-lateral neurons project antero-laterad (in direction to the lateral somata) (Fig. 3d). In one investigated specimen, the course turned further postero-mediad and finally to the longitudinal immunoreactive bundle (not shown). The neurites of the two posterior neurons possess several branchings while the main neurite crosses the midline in the posterior portion of the ganglion (Fig. 3e, h). The contralaterad projecting neurite bifurcates into an anteriad and a posteriad projection joining the longitudinal immunoreactive bundle (the same one as the lateral neurons) (Fig. 3e). The somata of the postero-medial neurons are located near the midline (Fig. 3f). The neurite of the left soma projects to the right hemiganglion, thus the projection pattern can be interpreted as contralateral (Fig. 3f). However, given its such close proximity to the midline, this classification is ambiguous.

### *Serotonin immunoreactivity in the ventral nerve cord of* Lithobius forficatus

**Fig. 4:**
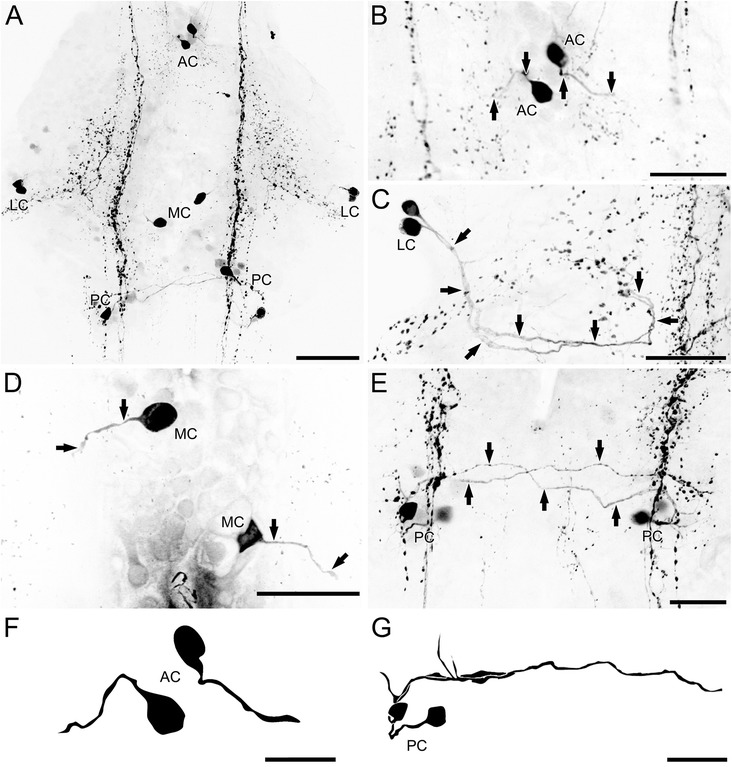
Distribution of 5HT-ir neurons in the VNC of *Lithobius forficatus*. All neurons were detected in every ganglion of all preparations. **a** These individually identifiable neurons are distinguished according to somata positions within the ganglia into anterior (AC), lateral (LC), medial (MC), and posterior cells (PC). **b** The AC projected their neurites contralaterally (arrows). **c** The LC project their neurites slightly posteriorly and medially towards the midline, and turn anteriorly again before the microscopic signal got lost (arrows). **d** Neurites of the medial cells could only be traced for a short distance (arrows), projecting laterad. **e** The pair of PCs project their processes contralaterally (arrows). **f** Reconstructions of ACs. **g** Reconstructions of PCs. Compare also Fig. 6. Scale bars: A = 100 μm; B–E, G = 50 μm; G = 25 μm

In *Lithobius forficatus*, four soma groups can be distinguished according to their spatial position of somata: an anterior (AC, Fig. 4a, b, f), two lateral (LC, Fig. 4a, c), two posterior (PC, Fig. 4a, e, g), and a single medial neuron (MC, Fig. 4a, d) per hemiganglion. The first three groups occupy positions and exhibit projection patterns corresponding to those described in *Scutigera coleoptrata* (compare Figs. 3a–c, e and 4a–c, e). However, the neurite of the anterior neuron remains ipsilateral (Fig. 4b, f). A medial neuron is absent in *Scutigera coleoptrata* and *Scolopendra oraniensis* (see below). The projection of the primary neurite could only be followed for a short distance, projecting laterad (Fig. 4d). Postero-medial (as found in *Scutigera coleoptrata*) and postero-lateral neurons (as found in *Scutigera coleoptrata* and *Scolopendra oraniensis*) are absent in *Lithobius forficatus*.

### *Serotonin immunoreactivity in the ventral nerve cord of* Scolopendra oraniensis

**Fig. 5:**
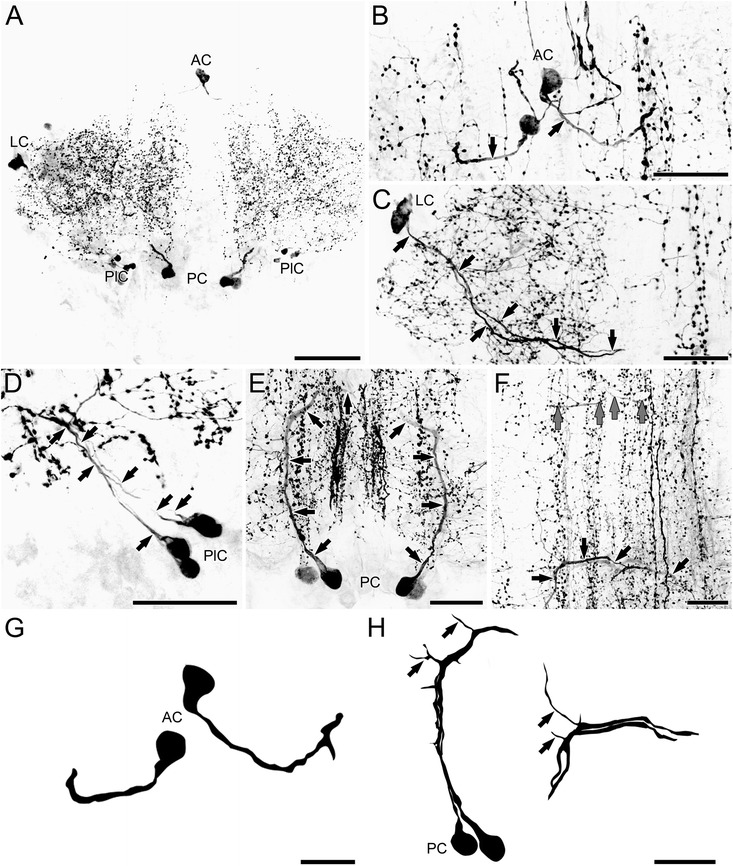
Distribution of 5HT-ir neurons in the VNC of *Scolopendra oraniensis*. All neurons were detected in every ganglion of all preparations. **a** These individually identifiable neurons can be distinguished according to somata positions within the ganglia into anterior (AC), lateral (LC), postero-lateral (PlC), and posterior cells (PC). **b** The initial projection of the ACs could been followed only for a short distance (arrows, but see f). **c** The LC project their neurites slightly posteriorly and medially towards the midline before the microscopic signal got lost (arrows). **d** Neurites of the PlC could only be traced for a short distance, projecting antero-laterally (arrows). **e** The pair of PCs project their processes anteriorly, before turning medially (arrows). **f** Contralateral projections could be detected in the anterior (gray arrows) and posterior parts (black arrows) of the ganglia at the level of the ACs and PCs, respectively. Compare also Fig. 6. **g** Reconstructions of ACs B. **h** Reconstructions of PCs (left) and its respective contralateral projections (right). Note that tracings were produced from different vibratome sections; arrows point to corresponding branching neurites. Compare also Fig. 6. Scale bars: A = 100 μm; B–F,H = 50 μm; G = 25 μm

In *Scolopendra oraniensis*, four groups of neurons are found in each hemiganglion: a single anterior neuron (AC, Fig. 5a, b, g), two lateral neurons (LC, Fig. 5a, c), two posterior neurons (PC, Fig. 5a, e, h), and four postero-lateral neurons (PlC, Fig. 5a, d). The first three groups are also found in similar positions and show projection patterns corresponding to those observed in *Scutigera coleoptrata* and *Lithobius forficatus* (except the ipsilateral projection of the anterior neuron in the latter; compare Figs. 3a–c, e, 4a–c, e, and 5a–c, e). Postero-lateral neurons could also be identified in *Scutigera coleoptrata*. However, in *Scolopendra oraniensis* this group comprises four neurons with smaller soma diameters (Fig. 5d, compare to Fig. 3d). Although the projections of the posterior neurons generally correspond to the course observed in the other species, they first project anteriad before crossing the midline (Fig. 5e, f, black arrows, h). Concerning the projection pattern of the anterior neurons, we were not able to follow their course as detailed as in *Scutigera coleoptrata*. However, we detected a 5HT-immunoreactive contralateral projection at the level of the anterior neurons in the adjacent slice, which most likely corresponds to the primary neurites of the anterior neurons (Fig. 5f, gray arrows). Neither a postero-medial neuron, as in *Scutigera coleoptrata*, nor a medial neuron, as in *Lithobius forficatus,* was detected in *Scolopendra oraniensis*.

**Fig. 6:**
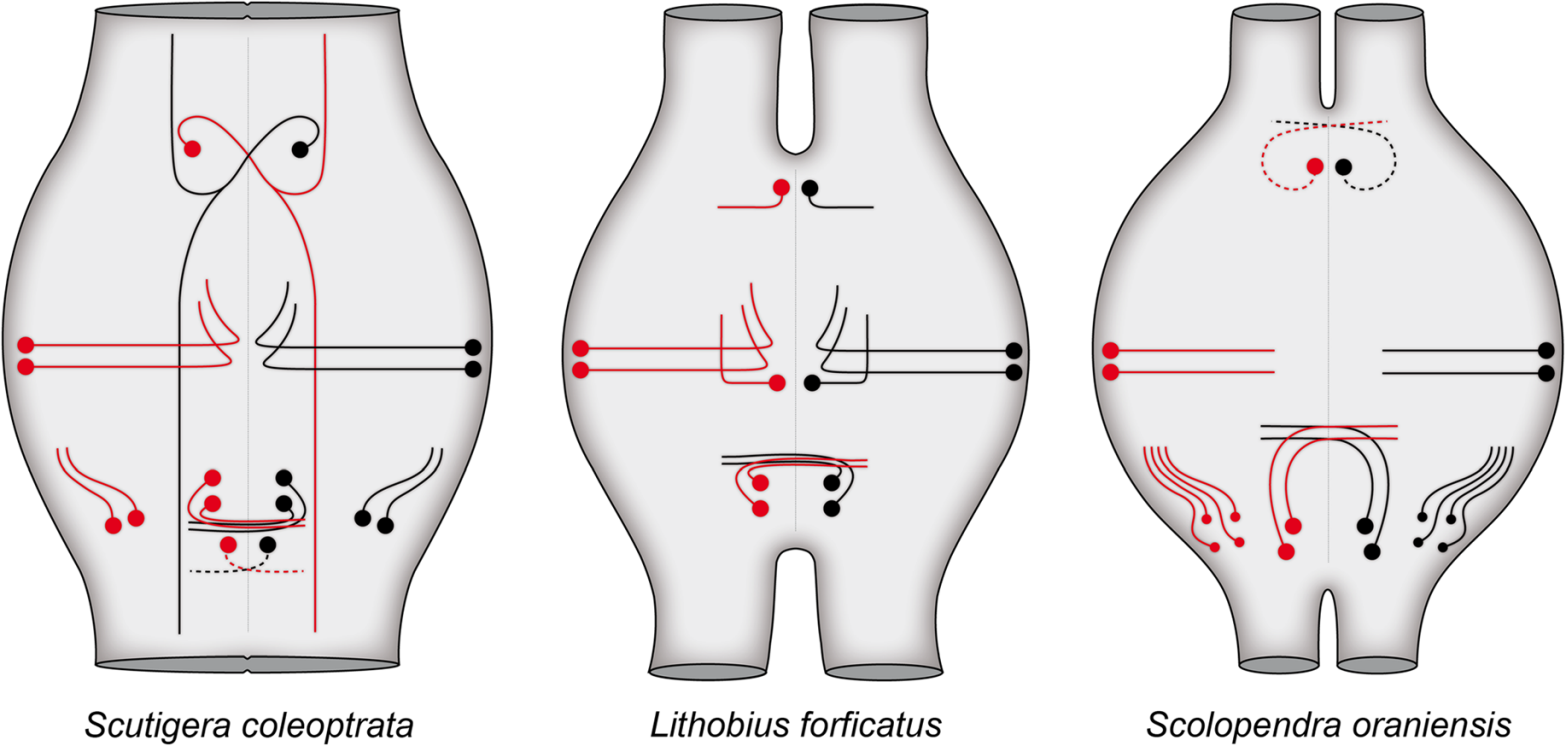
Schematic representations of the morphologies of the serially homologous 5HT-ir neurons in the investigated species. Neurons and the associated projections are depicted in red for the left hemiganglion, and in black for the right hemiganglion

A summary of the neuron sets and the major projections in the three investigated species is given in Fig. 6.

## Discussion

### The ventral nerve cord of Chilopoda

Our results on the general morphology of VNC ganglia are in concordance with previous studies [38–41]. In Lithobiomorpha and Scolopendromorpha distinct and separated connectives are present. In both species connectives are associated with only few somata compared to the soma cortex of ganglia (Fig. 2e, f). As a completely fused VNC was also described for the scutigeromorph centipede *Thereuopoda clunifera* [38], it can be assumed that this feature is an apomorphic character in Scutigeromorpha. In addition, Fahlander [38] pointed out that in Scutigeromorpha connectives possess a distinct cortex of somata. This feature is, however, ambiguous in *Scutigera coleoptrata*, as a clear separation between ganglion and medially fused connectives is not easily possible (compare Fig. 2a, d). Nevertheless, density of somata in the fused connective area appears equal to that surrounding the ganglia, which is in contrast to *Lithobius forficatus* and *Scolopendra oraniensis* (compare Fig. 2d–f).

### Serotonin-immunoreactive neurons in Chilopoda

In the three species investigated, the 5HT-ir system is strongly conserved within each ganglion of the ventral nerve cord and the detected neurons can be considered as serially homologous (Fig. 6). In one previous study, adult specimens of *Lithobius forficatus* and *Scolopendra* sp. were investigated [22]. Four groups of immunoreactive somata were identified and termed b, c, d, and e [22]. Most of these groups could be recovered in our re-investigation, but some differences are evident (see Table 1). Although Harzsch [22] stated that anterior neurons are absent in Myriapoda, we consider ‘c neurons’ as homologous to anterior neurons in Tetraconata, based on their position within the ganglia and the contralateral projection in *Scutigera coleoptrata* and *Scolopendra oraniensis* (see also discussion below). In *Scolopendra oraniensis* we detected two additional neurons in a lateral position within each hemiganglion that had not been identified previously [22]. Moreover, in *Scolopendra* sp. Harzsch found a cluster of 2–3 small somata in a medial position that was not evident in our preparations (Table 1). These differences may be explainable by interspecific differences, as we investigated *Scolopendra oraniensis* and Harzsch [22] presumably *Scolopendra subspinipes,* but definitively not *S. oraniensis* (pers. comm. Steffen Harzsch).

**Table 1:** Number and position of 5HT-ir neurons in a hemiganglion of Chilopoda

	*Scutigera coleoptrata*	*Lithobius forficatus*		*Scolopendra oraniensis*	
	This study	This study	Harzsch, 2004 [22]	This study	Harzsch, 2004^a^ [22]
Anterior	1	1	1 (c)	1	1 (c)
Lateral	2	2	2 (b)	2	-
Postero-lateral	2	-	-	4	4 (b)
Posterior	2	2	2 (e)	2	2 (e)
Postero-medial	1	-	-	-	-
Medial	-	1	1 (d)	-	2–3 (d)

Small letters refer to established neuron groups by Harzsch [22]. ‘c’ neurons are here interpreted as anterior neurons. ^a^ *Scolopendra* sp. investigated by Harzsch [22], (presumably *Scolopendra subspinipes*)

### Comparative neuroanatomy of serotonin-immunoreactive neurons

In the following, we compare each of the identified groups of immunoreactive neurons in the VNC of Chilopoda to 5HT patterns from other arthropods and then consider these data in an evolutionary context. Our interpretations, concerning the putative homology of neurons is, besides the shared neurotransmitter 5HT, based solely on morphological criteria, namely somata positions and neurite morphologies [42]. It should be kept in mind that unequivocal homologization requires further investigations, ideally cell lineage tracing of neurons. For instance, cell lineage studies in grasshoppers and fruit flies have revealed that the posterior serotonergic neurons in these species are derived from neuroblast 7-3 [5, 8, 9, 43]. Unfortunately, the lineages of 5HT-ir neurons in other arthropod taxa remain unknown. However, a common plan for neuronal development has been suggested by Thomas et al. [44], providing a rationale for deriving cellular homologies in arthropods. Expression analyses of the transcription factors *even-skipped* and *engrailed* across arthropod species have lent further support for the homology of certain neurons and neuroblast rows [45]. Tracer studies in Amphipoda (Crustacea) linked cell lineages from individual neuroblasts to identified pioneer neurons [46], suggesting homology of neuroblasts and their lineages, at least for Crustacea and Hexapoda. However, myriapods do not possess neuroblasts, but groups of mainly postmitotic neural precursors [47].

#### Anterior neurons

**Fig. 7:**
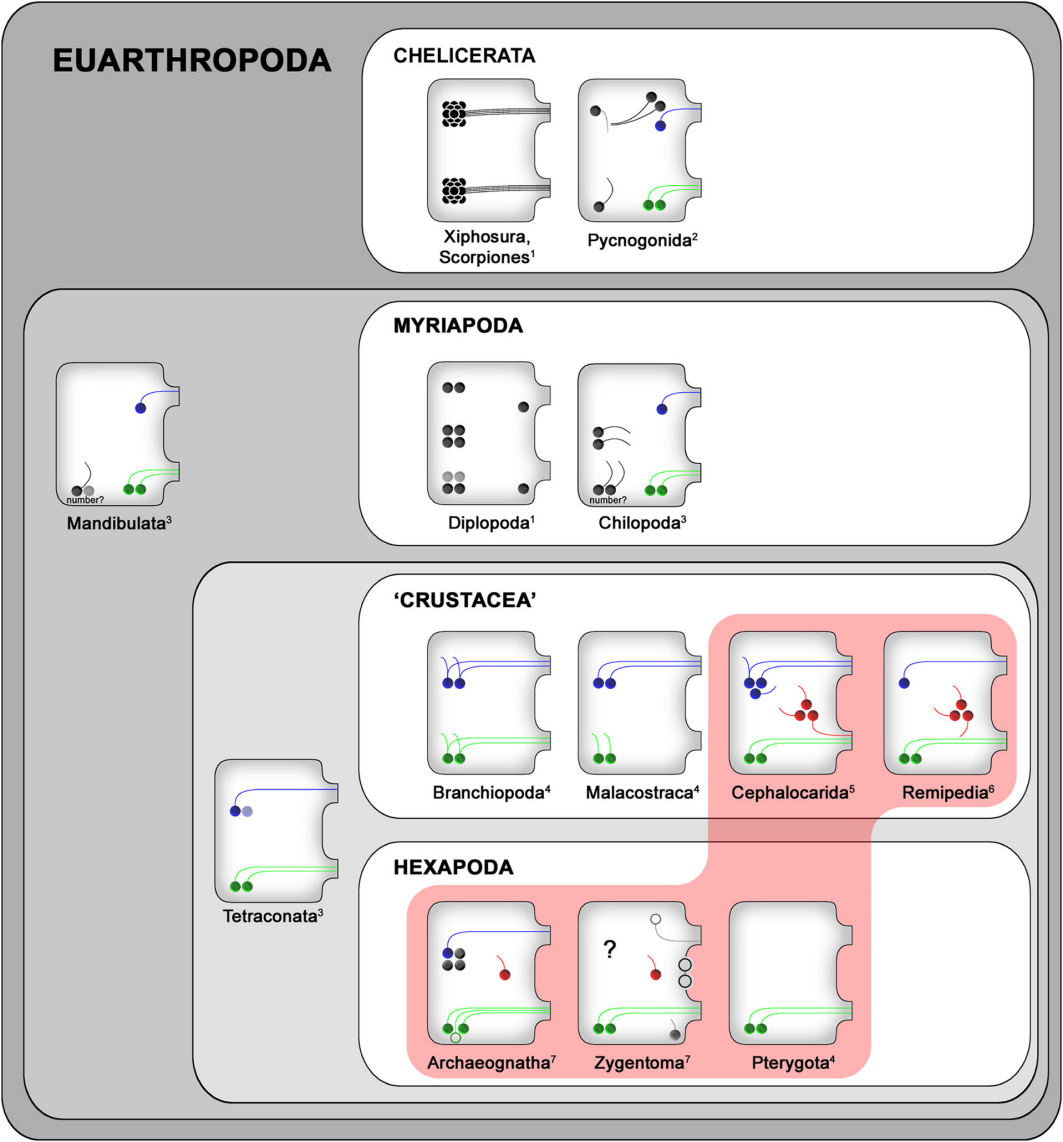
Patterns of 5HT-ir neurons in ganglia of the VNC for major euarthropod taxa. Blue, red, and green neurons indicate anterior, medial, and posterior somata, respectively. Gray neurons are those somata that cannot unequivocally be assigned to the ground patterns. Open circles in the archaeognathan and zygentoman patterns show neurons that are part of the thoracic but not of the abdominal pattern (see [1] for details). In Zygentoma, identification of anterior serially homologous neurons proved to be impossible due to intra- and interspecific variations in number, projection pattern and cell body size (marked by ‘?’, compare [1]). Red box encloses taxa, constituting the Miracrustacea (after [48]). Patterns modified from: ^1^ [22]; ^2^ [27]; ^3^ this study; ^4^ [21]; ^5^ [23]; ^6^ [24]; ^7^ [1]

We consider the projections of the anterior neurons in the centipede ground pattern as contralateral, as we identified contralateral projections in *Scutigera coleoptrata* (the most basal taxon investigated in the present study) and in *Scolopendra oraniensis* (Fig. 6). Consequently, we interpret the ipsilateral projection in *Lithobius forficatus* as apomorphic. Although number and projection patterns of anterior 5HT-ir neurons vary strongly between tetraconate taxa, a conspicuous congruence can be observed: In nearly all taxa, at least one anterior neuron exhibits a neurite projecting contralaterally (summarized in [23, 24]). In Chilopoda, the anterior soma is positioned antero-medially, near the midline, whereas in Tetraconata the soma is found far laterally (Figs. 6 and 7). Harzsch [22] proposed that from a mandibulate ground pattern, including anterior and posterior neurons with contralateral projections, in Chilopoda and Diplopoda anterior neurons were reduced and that antero-medially positioned somata are not homologous to anterior neurons in Crustacea and Hexapoda. However, due to the conspicuous neurite morphology, we suggest homology of the centipede medially-positioned anterior neurons with the tetraconate laterally-positioned anterior neurons and conclude one anterior neuron with a single neurite projecting contralaterally to be a general feature of Mandibulata (Fig. 7). In Diplopoda, Harzsch [22] detected antero-medially positioned somata with ipsilateral projection and homologized these to ‘c neurons’ in Chilopoda, but also antero-lateral neurons (‘a neurons’). As no data on neurite projections in Diplopoda are available, the question of whether ‘a neurons’ in Diplopoda are an apomorphic character remains open (this includes interpretation of all other neurons in Diplopoda as well, and will therefore not be considered in the discussion below). In Pycnogonida, an antero-medially positioned neuron with a single contralaterally projecting neurite is also present [27].

#### Medial neurons

As discussed in detail by Stemme et al. [1], the presence or absence of medial neurons in the centipede pattern is of special interest in the discussion of tetraconate phylogeny. In Tetraconata, these neurons are only found in the ground pattern of Cephalocarida, Remipedia, and basal hexapods (Fig. 7) but some faintly labeled neurons have been mentioned in several studies dealing with pterygote representatives [e.g. 1,4,8,13]. Unfortunately, the investigation of centipede species was not able to elucidate the evolution of these neurons, as one single medial pair of neurons could be detected in *Lithobius forficatus*, but is absent in *Scutigera coleoptrata* and *Scolopendra oraniensis*. This interpretation is further complicated by the presence of 2–3 smaller medially positioned neurons in *Scolopendra* sp. [22]. Interestingly, a neuron in similar position with a short ipsilateral projection was identified inconsistently in Pycnogonida [27].

#### Lateral neurons

Within euarthropods, immunoreactive somata in a lateral position have so far only been described in representatives of Myriapoda (Fig. 6). Harzsch [22] mentioned up to four neurons in a lateral position with unknown projection pattern within Diplopoda. We conclude that lateral 5HT-ir neurons are apomorphic at least in Chilopoda, and possibly Myriapoda.

#### Posterior neurons

The pair of posterior neurons with their contralateral projections identified in all three studied species strongly resembles those found in Tetraconata and Pycnogonida (Figs. 6 and 7). The only exceptions of this pattern are found in the crustacean taxa Branchiopoda (ipsi- and contralateral projections) and Malacostraca (only ipsilateral projections). One difference between Chilopoda and Pycnogonida to Tetraconata was observed: The somata of corresponding neurons are positioned laterally in Tetraconata, but more medially in Chilopoda and Pycnogonida. This complicates the homologization of neurons, as we additionally identified immunoreactive neurons in a postero-lateral position (also discussed by [27]). Thus, the tetraconate postero-lateral neurons might be homologized with two groups of immunoreactive neurons in Chilopoda: (1) the postero-lateral neurons found in *Scutigera coleoptrata* and *Scolopendra oraniensis* (Figs. 2d and 4d) correspond in soma position to the posterior neurons in Tetraconata, or (2) the pair of posterior neurons found in all three centipede species corresponds in number and projection pattern to the pair of posterior 5HT-ir neurons in lateral position in Tetraconata. In our opinion, the latter hypothesis is more parsimonious, as nearly every species investigated possesses a pair of posteriorly positioned contralaterally projecting neurons (Fig. 7). Thus, we suggest homology of the two contralaterally projecting neurons in Chilopoda and Pycnogonida to the more lateral pair in Tetraconata, undergoing a shift from a medial to a lateral position in the course of tetraconate evolution. In the blowfly *Calliphora erythrocephala*, somata of the posterior neurons in the meso- and metathoracic ganglion are relocated in the course of pupal development from a lateral to a more medial position [48]. Whether a similar developmental relocation of somata is evident in Chilopoda must be addressed in the future, as at present no developmental data are available for the investigated taxa.

#### Postero-lateral neurons

The number of postero-lateral immunoreactive neurons varies strongly between the three studied species (Fig. 6). Neurons in a corresponding position with similar neurite morphology have been described in Pycnogonida [27]. Here, one postero-lateral neuron with a short anteriad projection has consistently been observed. Within Tetraconata, no corresponding neurons have been described. However, a pair of neurons with similar position and projection pattern is part of the malacostracan pattern ([21]; Fig 7). Yet, it has been suggested that these neurons are homologous to the pair of contralateral projecting neurons in other tetraconate taxa [1, 21, 22, 24] (green neurons in Fig. 7).

#### Postero-medial neurons

We identified a single immunoreactive neuron in ganglia of *Scutigera coleoptrata*. Neurons in a corresponding position have been described in Zygentoma [1], but were only found inconsistently and could not be unequivocally assigned to the ground pattern [1]. Thus, homology of these neurons in *Scutigera coleoptrata* and Zygentoma remains unlikely and might represent apomorphies of both taxa.

### Reconstruction of a centipede ground pattern

Three corresponding groups of neurons have been found in each hemiganglion in all three studied species and are thus considered as interspecifically conserved: (1) an anterior neuron with a contralateral projection, (2) two lateral neurons with mediad ipsilateral projections, and (3) two posterior neurons with single contralateral projections (Fig. 7). Furthermore, two species (*Scutigera coleoptrata* and *Scolopendra oraniensis*) exhibit neurons in a postero-lateral position with ipsilateral projections, but the sizes of somata and numbers of neurons vary. Hence, it is reasonable to assign (4) neurons in a postero-lateral position to the ground pattern, although the number is not fixed (Fig. 7). Two categories of neurons, namely medial and postero-medial neurons, are only found in one species each. Thus, they are probably not part of the centipede ground pattern and instead reflect autapomorphies of the respective taxa.

## Conclusions

Our data support the following scenario for Tetraconata: The anterior (blue in Fig. 7) and posterior (green in Fig. 7) neurons have been retained from the mandibulate ground pattern, however, the position of the somata shifted from medial to lateral. The primary neurites of both groups of somata project contralaterally. As most tetraconatan representatives possess more than one anterior neuron (except for Remipedia and Hexapoda), at least a second anterior neuron with unknown projection pattern appears possible. The question of medial neurons (red in Fig. 7) in the tetraconate ground pattern [1, 23] could not be answered satisfactorily. Based on the current data, medial neurons thus would not be part of a tetraconate ground pattern, but would favor the ‘Miracrustacea hypothesis’, uniting Remipedia, Cephalocarida and Hexapoda (Fig. 7) [1, 49, 50]. However, a denser taxon sampling may reveal that medial neurons are more commonly distributed in Myriapoda, and may thus also be part of the ground pattern of Mandibulata. The similarities of the proposed myriapod, crustacean, hexapod, as well as the pycnogonid ground pattern (but a clear chelicerate pattern is ambiguous) thus suggest a mandibulate ground pattern containing an anterior and two posterior neurons with contralateral projections, with a soma positioned near the midline (Fig. 7). In addition, at least one neuron in postero-lateral position with a short ipsilateral projection is part of the mandibulate ground pattern (Fig. 7).

In Onychophora, no individually identifiable neurons in the VNC are detectable, as numerous serotonergic neurons are distributed rather randomly throughout the trunk ganglia [35, 51]. In Chelicerata, instead of individually identifiable neurons, clusters of up to 100 neurons have been described for larval *Limulus polyphemus* [22, 25] and scorpions [22, 36]. However, in the ventral nerve cord of the harvestman *Rilaena triangularis*, four 5HT-ir neurons per hemiganglion in ventro-medial position with ipsilateral projections were found [52]. Brenneis and Scholtz [27] showed individually identifiable serotonergic neurons in anterior and posterior position in walking leg ganglia of Pycnogonida. These data thus argue against a proposed plesiomorphic two-cluster ground pattern with contralateral neurite projections and variable numbers in Chelicerata, as individually identifiable 5HT-ir neurons with constant numbers are present in Opiliones and Pycnogonida. Independent evolution in both taxa seems unlikely [27].

Along these lines, homologization of 5HT-ir neurons of Pycnogonida and Mandibulata remains challenging. Although it is tempting to propose an euarthropod ground pattern of 5HT-ir neurons based on data of Pycnogonida, Chilopoda and various representatives of Tetraconata, character interpretation and polarization is difficult. Brenneis and Scholtz [27] listed correspondences of serotonergic neurons in Pycnogonida and Myriapoda based on the study by Harzsch [22], namely (1) a segmental set of few somata with similar numbers per hemiganglion, and (2) a stereotypic position of somata in anterior, posterior, lateral and medial positions. However, we argue against a homologization of lateral and medial neurons, as lateral neurons (by our definition, see above) are absent in Pycnogonida and medial neurons with ipsilateral projection were only found inconsistently in Pycnogonida and in the centipede *Lithobius forficatus*. We agree that individually identifiable serotonergic neurons in the ventral nerve cord certainly evolved in a common euarthropod ancestor but to further address homology assumptions, more studies on a variety of chelicerate as well as progoneate (Diplopoda, Symphyla and Pauropoda) taxa are needed. Conclusively, our comprehensive descriptions of the 5HT-ir system in the ventral nerve cord of Chilopoda provides a solid basis for such a survey.
